# Triple therapy revolutionizes treatment paradigms for previously untreatable HCC complicated by high-flow hepatic arteriovenous fistulas

**DOI:** 10.3389/fimmu.2025.1643290

**Published:** 2025-08-01

**Authors:** Jinpeng Li, Yuanming Li, Jingtao Zhong, Jiasheng Du, Jiao Chen, Jutian Shi, Lujun Zhao, Jinlong Song

**Affiliations:** ^1^ Department of Radiation Oncology, Tianjin Medical University Cancer Institute and Hospital, National Clinical Research Center for Cancer, Tianjin Key Laboratory of Cancer Prevention and Therapy, Tianjin’s Clinical Research Center for Cancer, Tianjin, China; ^2^ Department of Interventional Therapy I, Shandong Cancer Hospital and Institute, Shandong First Medical University and Shandong Academy of Medical Sciences, Jinan, Shandong, China; ^3^ Interventional Vascular Department, Laizhou Hospital of Traditional Chinese Medicine, Laizhou, Shandong, China; ^4^ Department of Hepatobiliary Surgery, Shandong Cancer Hospital and Institute, Shandong First Medical University and Shandong Academy of Medical Sciences, Jinan, Shandong, China; ^5^ Graduate Department of Shandong First Medical University and Shandong Academy of Medical Sciences, Jinan, China

**Keywords:** hepatocellular carcinoma, high-flow hepatic arteriovenous fistula, hepatic arterial infusion chemotherapy, immune checkpoint inhibitors, tyrosine kinase inhibitors

## Abstract

**Purpose:**

To evaluate the short-term efficacy and safety of hepatic arterial infusion chemotherapy (HAIC) combined with immune checkpoint inhibitors (ICIs) and tyrosine kinase inhibitors (TKIs) in patients with hepatocellular carcinoma (HCC) complicated by high-flow hepatic arteriovenous fistula (HAVF).

**Patients and methods:**

We retrospectively analyzed clinical data from 40 patients with unresectable HCC complicated by high-flow HAVF who received FOLFOX regimen HAIC plus ICIs and TKIs between January 2021 and June 2023. The efficacy evaluation included HAVF effective rate, tumor response, progression-free survival (PFS), overall survival (OS) per RECIST 1.1 and mRECIST. Adverse events (AEs) were recorded for safety evaluation.

**Results:**

The median follow-up time was 10.5 months (range: 3.5-16.4 months). A total of 150 HAIC cycles were administered, with a median frequency of 3.8 cycles per patient. The objective response rate (ORR) and the disease control rate (DCR) was 42.5% and 92.5% according to the RECIST 1.1, and 75.0% and 92.5% according to mRECIST criteria, respectively. The median PFS and the median OS were 5.5 months (95% CI: 3.9-6.9) and 10.4 months (95% CI: 7.4-13.4), respectively. In univariate analysis, HAVF grade, extrahepatic spread, HAVF disappearance were potential prognostic factors for OS, while HAVF grade and extrahepatic spread being independently associated with PFS. Hypertension (12.5%), Thrombocytopenia (12.5%) and Albumin decreased (7.5%) were the most frequently observed grade 3-4 TRAEs.No treatment-related mortality occurred during the study period.

**Conclusion:**

HAIC combined with ICIs and TKIs demonstrates promising short-term efficacy and acceptable safety in patients with unresectable HCC complicated by high-flow HAVF. This combination therapy effectively controls tumor growth while simultaneously managing the arteriovenous shunt, providing a valuable treatment option for this challenging patient population.

## Introduction

Hepatocellular carcinoma (HCC) is one of the most common malignant tumors worldwide and a leading cause of cancer-related death (1). Approximately 10~15% of HCC patients develop high-flow hepatic arteriovenous fistulas (HAVF), including arterioportal shunts (APS) and arteriovenous shunts (AVS) (2, 3). HAVF not only promotes rapid tumor progression but also risks inducing life-threatening complications such as portal hypertension, esophageal variceal hemorrhage, and hepatic encephalopathy, significantly shortening patient survival (4).

Transarterial chemoembolization (TACE) is widely recognized as a standard treatment option for patients with unresectable HCC (5). However, in HCC patients with high-flow hepatic arteriovenous fistulas, the application of TACE faces significant challenges. The altered hemodynamics in these patients complicates TACE procedures, increasing technical difficulty and the risk of complications (6).These factors collectively limit the application value and clinical benefit of TACE in this population.

Hepatic arterial infusion chemotherapy (HAIC) has experienced growing utilization in managing unresectable HCC over recent years, and the oxaliplatin-based FOLFOX regimen has been adopted as the standard first-line treatment in China (7). Studies have confirmed that FOLFOX-HAIC significantly extends survival compared to sorafenib in patients with advanced HCC (8). For HCC with high-flow hepatic arteriovenous fistulas, HAIC offers unique advantages: locally concentrated drug delivery enhances antitumor effects, promotes significant tumor shrinkage facilitating fistula closure, and standardized procedures reduce the risk of complications (6, 9). These features make HAIC an ideal option for these patients. However, HAIC monotherapy has shown limited efficacy. Given the breakthroughs achieved with immune checkpoint inhibitors and targeted agents in HCC treatment (10, 11), the strategy of combining HAIC with these emerging therapies has gained considerable attention. This multimodal approach may further improve efficacy through synergistic effects, though its clinical value in HCC patients with high-flow hepatic arteriovenous fistulas requires systematic evaluation.

The objective of this study was to evaluate the efficacy and safety of HAIC in combination with ICIs and TKIs for treating patients with unresectable HCC complicated by high-flow hepatic arteriovenous fistulas. By conducting a retrospective analysis of cases from our center, we sought to enhance therapeutic strategies for these difficult-to-treat patients and create a foundation for upcoming prospective studies.

## Methods

### Patient selection

**Figure 1:**
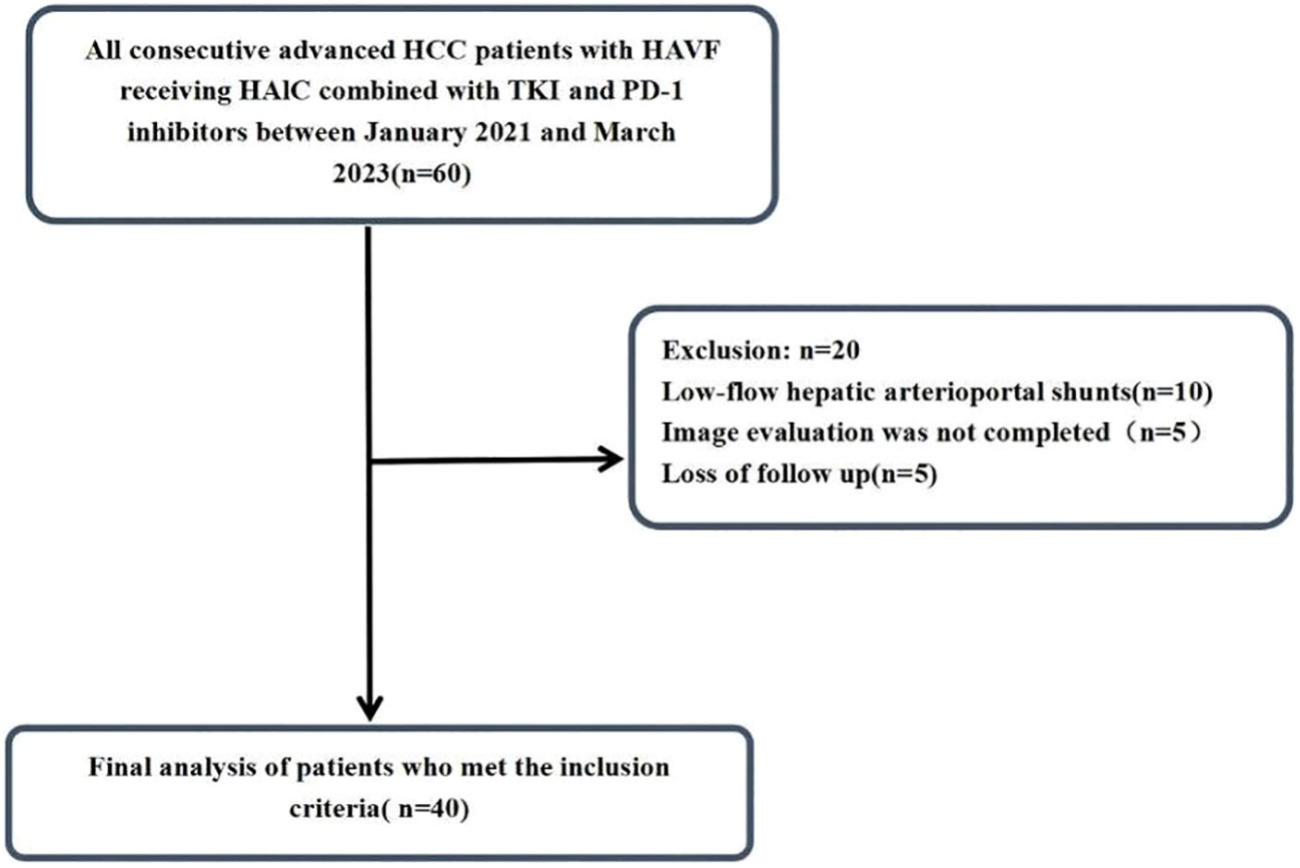
Flow chart.

The study design and analytical approach are schematically represented in **Figure 1**. Initially, 60 patients who were diagnosed with hepatocellular carcinoma (HCC) complicated by hepatic arteriovenous fistula at Shandong Cancer Hospital and Institute from January 2021 to June 2023 were identified for this study. After applying the inclusion and exclusion criteria, 40 HCC patients were ultimately included. The medical record data of the patients were collected through retrospective analysis. This study was approved by the Ethical Review Committee of the Shandong Cancer Hospital and Institute. Informed consent was waived because this study was retrospective. All data were anonymized and handled in strict compliance with confidentiality regulations. This study was conducted in accordance with the Declaration of Helsinki.

### HAVF classification

The diagnosis of HCC was rigorously confirmed in all cases using either well-established American Association for the Study of Liver Diseases (AASLD) or the European Association for the Study of the Liver (EASL) (12), or through conclusive pathological examination of liver tissue. Hepatic arteriovenous fistulas (HAVF), comprising arterial-portal shunts (APS) and arterial-hepatic venous shunts (AVS), can be diagnosed and classified through DSA or contrast-enhanced CT (13, 14). HAVF evaluation and severity classification were accomplished via DSA imaging, incorporating a standardized grading approach based on existing studies (15–17), as presented, as follows: The severity grading employs a 4-level system: grade 0 indicates negligible or minimal restrictive shunting; grade 1 represents shunting affecting local branches (APS to segmental portal vein, AVS to local hepatic venous branches); grade 2 was assigned to shunt reaching the ipsilateral main trunk (APS to ipsilateral main portal vein, AVS to hepatic segmental vein); grade 3 was defined for cases where the shunt reached the main portal vein presenting with hepatofugal portal venous flow shunting affecting contralateral or major veins (AVS to main hepatic vein or inferior vena cava) (6, 17, 18). Both Grade 2 and 3 were classified as high-flow fistulas.

### Inclusion criteria and exclusion criteria

Patients were carefully selected based on the following inclusion criteria (1): age 18 years or older; (2) preserved liver function with Child-Pugh A-B or ALBI grade 1-2; (3) good performance status (ECOG 0-1); (4) presence of high-flow HAVF (grade 2-3) documented by DSA or contrast-enhanced CT; (5) ineligibility for curative surgical resection or ablative procedures; and (6) expected survival of at least 3 months. Patients were excluded if they had (1) incomplete clinical records; (2) insufficient follow-up (<6 months); (3) prior HAIC or systemic treatment; (4) complete main portal vein occlusion with inadequate collateral flow; or (5) concurrent malignancies.

### HAIC

All patients underwent detailed superior mesenteric and hepatic angiography for comprehensive tumor and HAVF evaluation. The femoral artery was punctured using standard Seldinger technique under local anesthesia, with a 5 French catheter subsequently deployed to analyze hepatic arterial configuration and tumor blood supply patterns. Under Digital Subtraction Angiography (DSA) or Cone Beam CT (CBCT) guidance, the operator performed angiography of the celiac trunk, superior mesenteric artery, and infra-diaphragmatic arteries to comprehensively evaluate tumor vascularity. Using guidewire support, a microcatheter was precisely positioned within the main arterial feeders supplying the tumor. Collateral circulation was selectively embolized beforehand to redirect blood flow and improve therapeutic delivery to the target lesion. Any flow detected in the gastroduodenal artery or remote extrahepatic vessels was effectively blocked using coil embolization. After catheter placement, heparinized saline (10,000 U heparin in 100 mL 0.9% sodium chloride) was administered to prevent intracatheter thrombosis. The treatment schedule utilized modified FOLFOX6, featuring oxaliplatin (85 mg/m² delivered over the first 2 hours of day 1), leucovorin (400 mg/m² given during hours 2-4 of day 1), and fluorouracil (400 mg/m² bolus at hour 4, then 2,400 mg/m² as a 46-hour continuous infusion on days 1-2). HAIC treatments continued every 3 weeks until progression or prohibitive toxicity emerged. Dose reductions were acceptable based on hepatic reserve and drug tolerance, adhering to guidelines from prior studies (7, 19).Throughout treatment, patients underwent close monitoring of vital signs and laboratory parameters, with regular imaging assessments. For all HBV-infected patients, baseline viral load testing was performed, and effective antiviral management was implemented when necessary.

### Systemic treatment

TKIs and PD-1 inhibitors were initiated at least 3 days before or after HAIC and maintained until disease progression or prohibitive toxicity developed. Before treatment, patients were fully briefed on therapeutic efficacy and potential adverse events. PD-1 inhibitors were delivered intravenously every 3 weeks over 30-60 minutes at fixed doses: sintilimab 200 mg, camrelizumab 200 mg, and tislelizumab 200 mg. Tyrosine kinase inhibitors (TKIs), including apatinib (250 mg, once daily), donafenib (200 mg, once daily), and lenvatinib (8 mg, once daily), was administered orally. Treatment was halted in the event of any unacceptable or severe adverse event (AE) (grade 3 or higher AE) or any intolerable grade 2 drug-related AE. Treatment was also discontinued when disease progression was observed.

### Outcomes

Multiple clinical outcomes were investigated, including tumor response, HAVF improvement, HAVF recanalization, OS, PFS, and AEs. Based on Response Evaluation Criteria in Solid Tumors version 1.1 (RECIST v1.1) and Modified RECIST (mRECIST) standards, systematic tumor response evaluation was performed at 4-8 week intervals using MRI and dynamic contrast-enhanced CT to precisely monitor tumor burden changes and necrotic area distribution. The treatment response represented the optimal response observed throughout the follow-up period, categorized as complete response (CR), partial response (PR), progressive disease (PD), or stable disease (SD).The objective response rate (ORR) was determined as the combined total of CR and PR, while the disease control rate (DCR) was computed as the cumulative count of CR, PR, and SD.PFS was calculated as the period from the start of combination therapy to disease progression or death), and OS was defined as the time between the start of the therapy and death from any cause. Improvement in HAVF was a reduction grade one level, while no improvement or progression of the fistula after treatment was deemed ineffective. Adverse events were documented and evaluated according to the Common Terminology Criteria for Adverse Events version 5.0.

### Data processing and analysis

Statistical analyses were performed using SPSS software (version 26.0). Parametric continuous variables were expressed as mean ± standard deviation and evaluated through Student’s t-test, whereas non-parametric continuous variables were reported as medians and assessed via Mann-Whitney U-test. Categorical variables were described as counts and percentages, with group comparisons performed using chi-square tests or Fisher’s exact tests when appropriate. Survival analysis was conducted using the Kaplan-Meier approach, with log-rank testing for intergroup comparisons. Both univariate and multivariate Cox proportional hazards analyses were implemented to determine prognostic indicators. Statistical significance was defined as a two-tailed p-value < 0.05.

## Results

### Patient characteristics

By the end of follow-up in August 2024, a total of 60 HCC patients met the inclusion criteria. Among them,10 patients with low-flow hepatic arterioportal shunts, 5 patients did not complete imaging examination and evaluation after treatment, 5 patient was lost to follow-up, all of which were excluded (**Figure 1**). Finally, 40 HCC patients were included in this study, and their baseline characteristics were summarized in **Table 1**. Male patients accounted for 72.5% of the study population. Cirrhosis was present in 62.5% of patients. Patients with Vp3-4 portal vein tumor thrombus accounted for 90%(36/40) of the cohort. Most patients (85.0%) were HBsAg positive. Extrahepatic metastasis was observed in 27.5% of patients. The majority of patients (95.0%) had Barcelona Clinic Liver Cancer (BCLC) stage C disease, while 5.0% had BCLC stage B disease. Regarding hepatic arteriovenous fistula (HAVF), 42.5% of patients were classified as grade 2 and 57.5% as grade 3. For HAVF somatotype, 32.5% of patients had arteriovenous shunt (AVS) and 67.5% had arterioportal shunt (APS).

**Table 1:** Basic clinical characteristics of the included patients.

Characteristics	Number of patients [n (%)]
Gender	
Male	29 (72.5%)
Female	11 (27.5%)
Age, years	57.00 (53.75- 61.00)^a^
HBsAg	
Positive	34 (85.0%)
Negative	6 (15.0%)
Cirrhosis	
Yes	25 (62.5%)
No	15 (37.5%)
AFP	
>400	25 (62.5%)
≤400	15 (37.5%)
Tumor Diameter	10.26 (3.62-15.6)^a^
Tumor number	
>3	25 (62.5%)
≤3	15 (37.5%)
Extrahepatic metastasis	
Yes	11 (27.5%)
No	29 (72.5%)
PVTT	
No	4 (10.0%)
Vp3	12 (30.0%)
Vp4	24 (60.0%)
BCLC stage	
B	2 (5.0%)
C	38 (95.0%)
Child-Pugh	
A	32 (80.0%)
B	8 (20.0%)
ALBI	
1	23 (57.5%)
2	16 (40.0%)
3	1 (2.5%)
ECOG	
0	10 (25.0%)
1	30 (75.0%)
HAVF classification	
2	17 (42.5%)
3	23 (57.5%)
HAVF type	
AVS	13 (32.5%)
APS	27 (67.5%)
ALT,u/L	36.30 (31.12-48.60)^a^
TBIL, umol/L	14.35 (11.57-25.70)^a^
HGB, g/L	135.00 (125.75-141.25)^a^
WBC, x10^9^/L	5.27 (4.25-6.79)^a^
PLT, x10^9^/L	136.50 (97.75-199.25)^a^
NLR	2.26 (1.58-3.70)^a^
PLR	103.52 (63.30-141.40)^a^
MTTs	
Lenvatinib	32 (80.0%)
Donafenib	8 (20.0%)

The data are presented as quantity (percentage), unless otherwise specified.

a The data are expressed as median (IQR).

AFP, alpha-fetoprotein; PVTT, portal vein tumor thrombus; BCLC, Barcelona clinic liver cancer; Child-Pugh, Child-Pugh Classification; ALBI, albumin-bilirubin score; ECOG PS, Eastern Cooperative Oncology Group performance status; HAVF, hepatic arteriovenous fistula; ALT, alanine aminotransferase; TBil, total bilirubin; HGB, hemoglobin; WBC, white blood cell count; PLT, platelet count; NLR, neutrophil-to-lymphocyte ratio; PLR, platelet-to-lymphocyte ratio; AVS, arteriovenous shunts; APS, arterioportal shunt; IQR, interquartile range; MTTs, molecular targeted therapies.

### Efficacy outcomes

**Figure 2:**
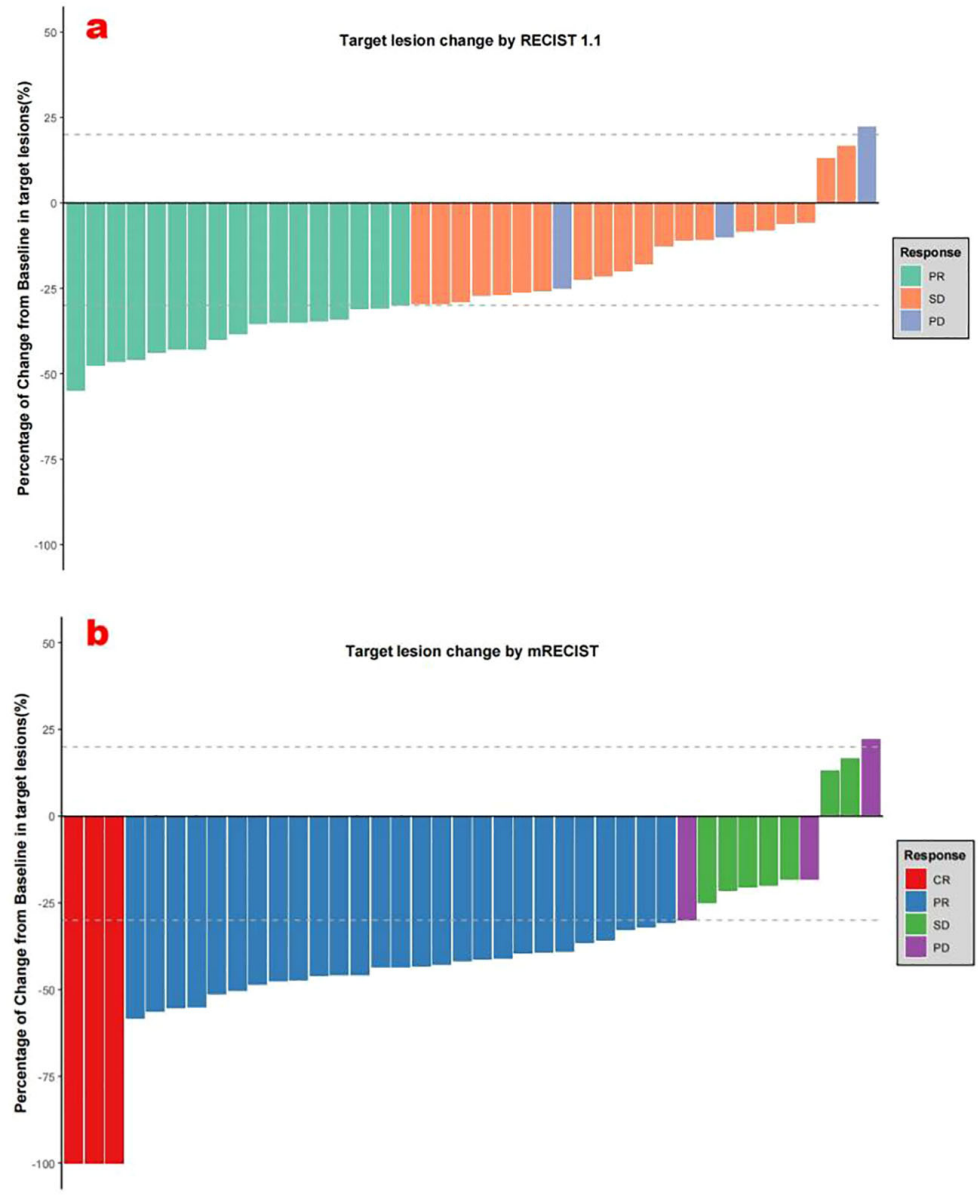
**(a)** Best percentage changes from baseline in target lesions per RECIST v1.1. **(b)** Best percentage changes from baseline in target lesions per mRECIST.

The follow-up duration reached a median of 13.5 months (3.5-16.4 months). During the study period, 150 HAIC treatment sessions were delivered, averaging 3.75 cycles per patient. According to mRECIST evaluation criteria, patients treated with HAIC+TKI+PD-1 inhibitor combination demonstrated CR in 7.5%, PR in 67.5%, and SD in 17.5% of cases, resulting in an ORR of 75.0% and DCR of 92.5%. When assessed using RECIST v1.1 criteria, the ORR and DCR were 42.5% and 92.5%, respectively (**Table 2**, **Figures 2a, b**).

**Table 2:** Tumor response assessment according to RECIST 1.1.

Tumor responses	RECIST1.1	mRECIST
CR	0	3
PR	17	27
SD	20	7
PD	3	3
ORR	42.5%	75.0%
DCR	92.5%	92.5%

CR, complete response; DCR, disease control rate; ORR, objective response rate; PD, progressive disease; PR, partial response; SD, stable disease; RECIST1.1,Response Evaluation Criteria in Solid Tumors1.1; mRECIST, Modified Response Evaluation Criteria in Solid Tumors.

**Figure 3:**
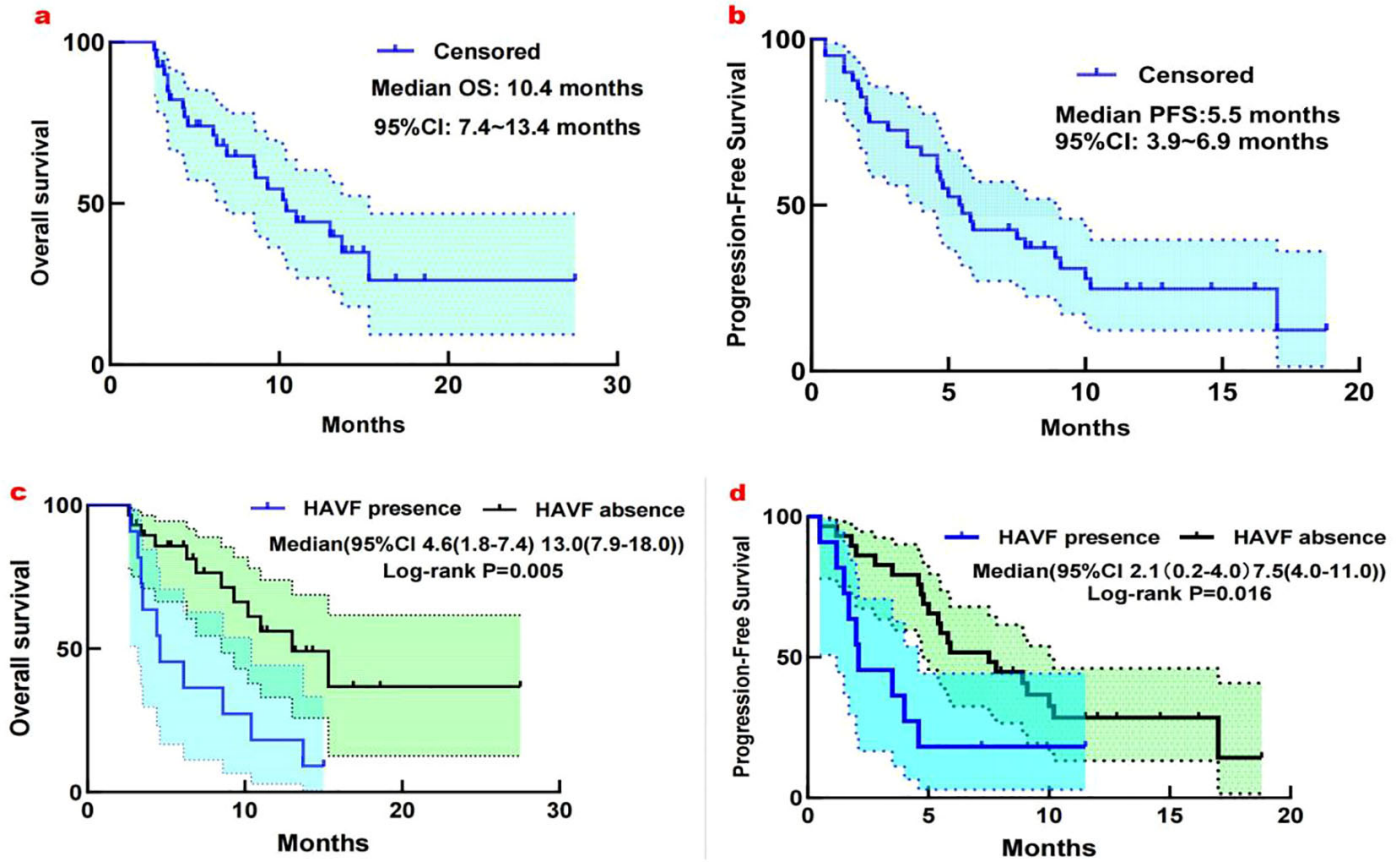
**(a)** Kaplan-Meier analysis of overall survival in 40 patients with unresectable HCC complicated by high-flow HAVF treated with HAIC plus TKI and PD-1 inhibitors. Median OS: 10.4 months (95% CI: 7.4-13.4 months). **(b)** Kaplan-Meier analysis of progression-free survival in 40 patients with unresectable HCC complicated by high-flow HAVF treated with HAIC plus TKI and PD-1 inhibitors. Median PFS: 5.5 months (95% CI: 3.9-6.9 months). **(c)** Subgroup analysis for overall survival considering the presence or absence of HAVF. **(d)** Subgroup analysis for Progression-free survival considering the presence or absence of HAVF.

During follow-up,18 OS events occurred, with a median OS of 10.4 months (95% CI: 7.4-13.4; **Figure 3a**) and a median PFS of 5.5 months (95% CI: 3.4–6.9; **Figure 3b**). Univariate analysis revealed several factors significantly associated with overall survival (OS), including extrahepatic metastasis (HR 0.46, 95% CI 0.23-0.92, p=0.030), BCLC staging (HR 1.98, 95% CI 1.09-3.58, p=0.024), HAVF disappearance (HR 0.31, 95% CI 0.17-0.55, p<0.001), and HAVF grade (HR 2.51, 95% CI 1.40-4.48, p=0.002). In the subsequent multivariate analysis, extrahepatic metastasis (HR 0.34, 95% CI 0.17-0.71, p=0.004), BCLC staging (HR 2.00, 95% CI 1.15-3.48, p=0.015), and HAVF disappearance (HR 0.29, 95% CI 0.16-0.55, p<0.001) remained independently predictive of OS.

For progression-free survival (PFS), univariate analysis demonstrated significant associations with extrahepatic metastasis (HR 0.52, 95% CI 0.30-0.89, p=0.019), HAVF disappearance (HR 0.68, 95% CI 0.52-0.89, p=0.005), HAVF grade (HR 3.19, 95% CI 1.66-6.11, p<0.001), and PVTT classification (HR 1.03, 95% CI 1.00-1.06, p=0.034). Multivariate Cox regression analysis confirmed extrahepatic metastasis (HR 0.56, 95% CI 0.32-0.99, p=0.045), HAVF disappearance (HR 0.20, 95% CI 0.05-0.83, p=0.027), and HAVF grade (HR 2.66, 95% CI 1.27-5.57, p=0.009) as independent prognostic indicators for PFS. Other clinical parameters including gender, AFP levels, tumor number, ECOG performance status, and Child-Pugh classification showed no statistically significant correlation with either survival endpoint (**Table 3**).

**Table 3:** Univariate and multivariate analyses of predictors of survival.

Characteristics	OS				PFS			
	Univariate analysis		Multivariate analysis		Univariate analysis		Multivariate analysis	
	HR (95% CI)	P	HR (95% CI)	P	HR (95% CI)	P	HR (95% CI)	P
Gender (male vs. female)	1.29 (0.18-9.47)	0.800			1.22 (0.53–2.79)	0.640		
AFP (>400 vs.<400)	1.18 (0.64-2.17)	0.600			1.30 (0.50-3.39)	0.590		
Tumor number (>3 vs. ≤3)	1.09 (0.52-2.32)	0.810			1.97 (0.47-8.15)	0.350		
Extrahepatic metastasis (No vs. yes)	0.46 (0.23-0.92)	0.030	0.34 (0.17-0.71)	0.004	0.52 (0.30-0.89)	0.019	0.56 (0.32-0.99)	0.045
BCLC (C vs. B)	1.98 (1.09-3.58)	0.024	2.00 (1.15-3.48)	0.015	1.32 (0.74-2.33)	0.350		
ECOG-PS (1 vs.0)	1.42 (0.67-2.99)	0.359			1.79 (0.85~3.77)	0.128		
HAVF disappearance (yes vs. no)	0.31 (0.17-0.55)	<0.001	0.29 (0.16-0.55)	<0.001	0.68 (0.52-0.89)	0.005	0.20 (0.05-0.83)	0.027
HAVF grade (Grade 3 vs. 2)	2.51 (1.40-4.48)	0.002	1.78 (0.85~3.77)	0.128	3.19 (1.66-6.11)	<0001	2.66 (1.27-5.57)	0.009
Child-Pugh (B vs.A)	1.613 (0.80~3.25)	0.181			1.30 (0.50-3.39)	0.589		
PVTT (VP4 vs.VP3)	1.87 (0.43~8.15)	0.400			1.03 (1.00-1.06)	0.034	1.42 (0.67~2.99)	0.359

AFP, a-fetoprotein; BCLC, Barcelona clinic liver cancer; ECOG PS, Eastern Cooperative Oncology Group performance status; PFS, progression-free survival; PVTT, portal vein tumor thrombus.

We analyzed how HAVF affected survival by examining outcomes in patients with and without HAVF during follow-up. Of patients presenting with HAVF, resolution occurred in 29 patients (72.5%), while HAVF persisted in 11 patients (27.5%). Significant survival differences emerged between groups. Patients without HAVF demonstrated superior survival metrics compared to those with HAVF (median OS: 13.0 months [95% CI, 7.9-18.0] vs 4.6 months [1.8-7.4], P=0.005; median PFS: 7.5 months [4.0-11.0] vs 2.1 months [1.3-4.9], P=0.016; **Figures 3c, d**). In multivariate analysis, HAVF grade, extrahepatic spread, and HAVF disappearance were found to be independent prognostic markers associated with PFS and OS (**Table 3**).

### Outcome of HAVF

**Figure 4:**
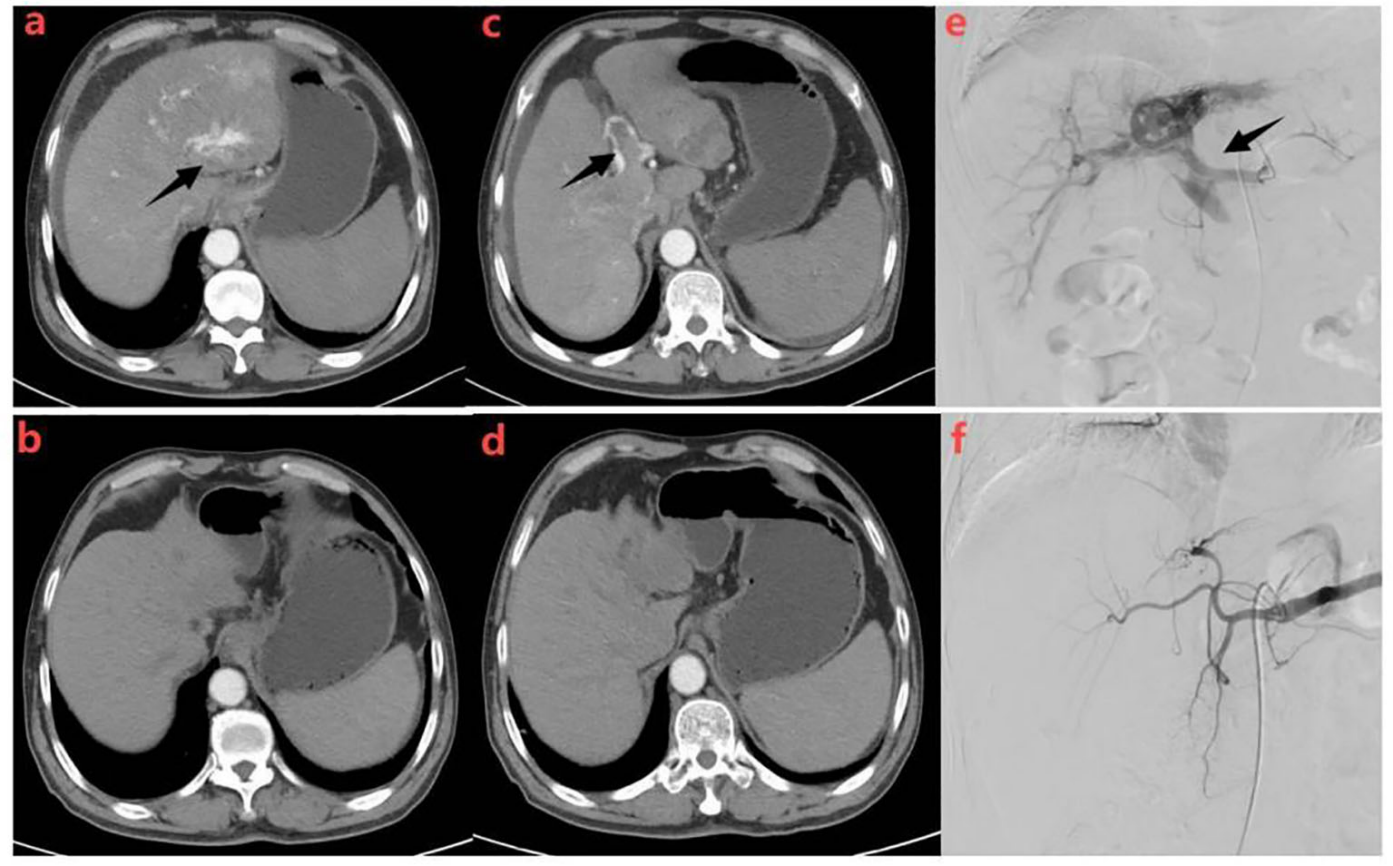
Representative radiographical images of a patient who achieved APS disappearance after treatment. Images of man aged 58 years with HCC complicated by Vp4 PVTT and severe APS after receiving triple therapy.Axial contrast-enhanced CT images in the arterial phase **(a)** and portal phase **(c)** before the initiation of treatment. CT images taken during the arterial phase **(a)** displayed early enhancement of portal vein branches (indicated by a black arrow), representing APS. Portal venous phase **(c)** showed filling defects in the main trunk and left branch of the portal vein, representing tumor thrombi in the portal vein (indicated by a black arrow).Hepatic angiography **(e)** revealed severe APS and Vp4 PVTT(indicated by a black arrow). Subsequent axial contrast-enhanced CT images in the arterial **(b)** and portal **(d)** phase were taken after three cycles of triple therapy, showing no apparent APS and regression of portal vein tumor thrombus. Hepatic angiography in image **(f)** confirmed the absence of APS.

**Figure 5:**
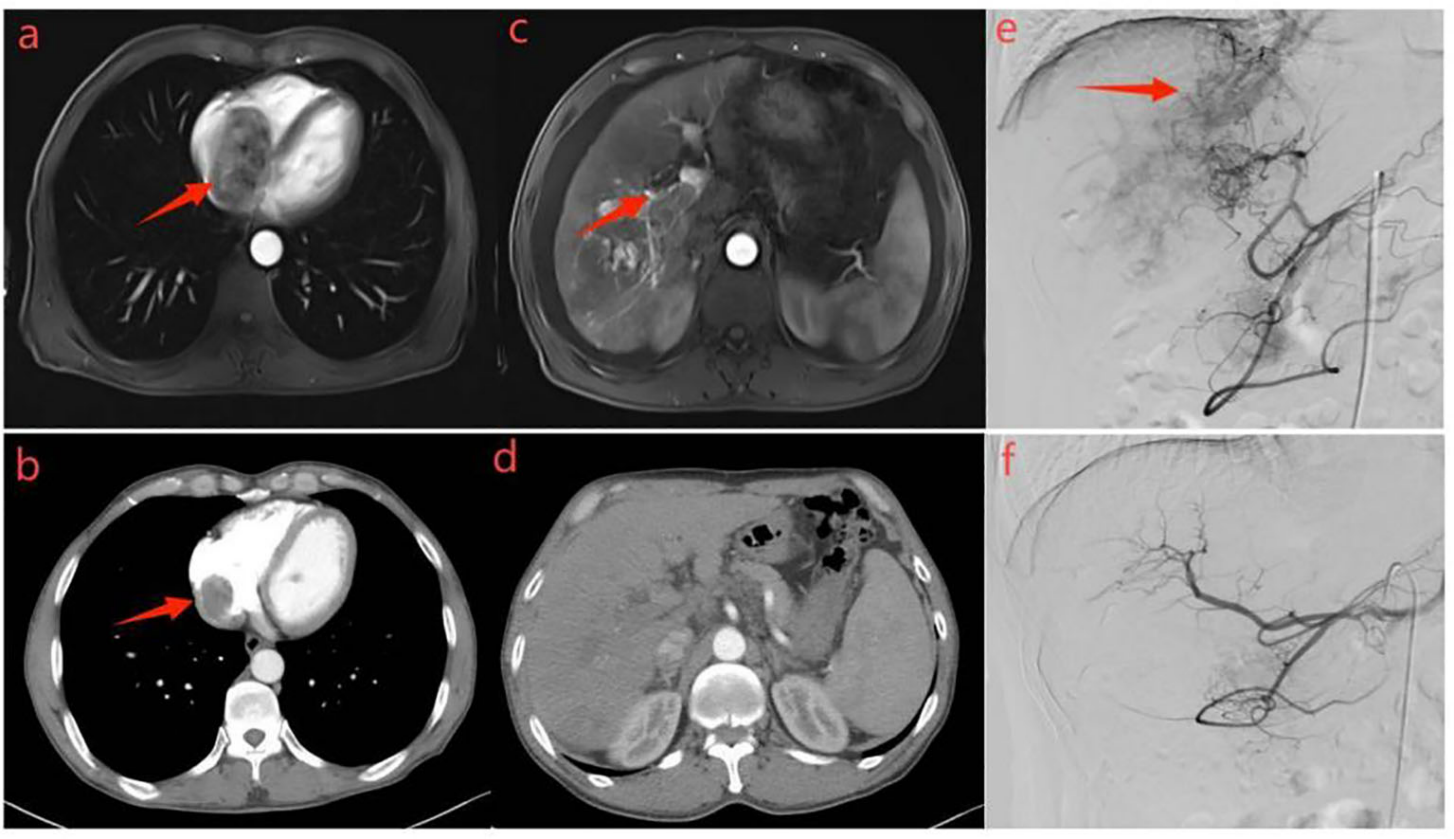
Representative radiographical images of a patient who achieved AVS disappearance after treatment. Images of man aged 65 years with HCC complicated by right atrial tumor thrombus and severe AVS after receiving triple therapy. Axial contrast-enhanced MRI images in the arterial phase **(a)** and portal phase **(c)** before the initiation of treatment. MRI images taken during the arterial phase **(a)** displayed early enhancement of tumor and right atrial cavity filling defect (indicated by a black arrow), representing AVS. Portal venous phase **(c)** showed filling defects in the main trunk and left branch of the portal vein, representing tumor thrombi in the portal vein (indicated by a black arrow).Hepatic angiography **(e)** revealed severe AVS and right atrial tumor thrombus (indicated by a black arrow). Subsequent axial contrast-enhanced CT images in the arterial **(b)** and portal **(d)** phase were taken after two cycles of triple therapy, showing no apparent APS and regression of right atrial tumor thrombus. Hepatic angiography in image **(f)** confirmed the absence of AVS.

After triple therapy, the efficacy rate of HAVF treatment reached 85.0%(34/40)(**Table 4**). The majority of patients demonstrated significant hemodynamic improvement following treatment intervention. Complete disappearance of HAVF was documented in 29 (72.5%) patients during follow-up imaging studies (**Figures 4**, **5**). These cases showed complete resolution of the abnormal arteriovenous communication with restoration of normal hepatic vascular architecture.

**Table 4:** Outcome of HAVF.

Characteristics	N (%)
HAVF improved	34/40 (85.0%)
HAVF disappeared	29/40 (72.5%)
Cancer thrombus reduced	31/36 (86.1)
Tumor reduced	17/40 (42.5%)
Cancer embolus reduced and HAVF improved	31/31 (100%)
Tumor reduced and HAVF improved	16/17 (94.1%)
HAVF recanalization	0/40 (0.00%)

The data are presented as quantity (percentage), unless otherwise specified.

HAVF, hepatic arteriovenous fistula.

Concurrent reduction in cancer thrombus was observed in 31 of 36 evaluable patients (86.1%), indicating effective management of the underlying malignant process. Tumor reduction was documented in 17 (42.5%) patients, reflecting the dual therapeutic benefit of the intervention on both vascular and oncologic outcomes.

Among patients presenting with both cancer embolus and HAVF, combined improvement was achieved in all 31 cases (100%). Furthermore, in patients with concurrent tumor burden and HAVF, combined improvement was observed in 16 of 17 cases (94.1%), demonstrating the efficacy of treatment in addressing multiple pathologic processes simultaneously.

Most importantly, recanalization events were completely absent among all patients during follow-up (0/40, 0.0%), indicating sustained therapeutic success. This absence of recurrence suggests that the treatment approach provides sustained vascular stabilization without compromise of long-term patency (**Table 4**).

### Safety and adverse events

Treatment-related mortality was not observed in this study. At least one treatment-related adverse event was reported in 35 patients (87.5%) (**Table 5**). Treatment- and laboratory-related adverse events (AEs) were evaluated according to CTCAE version 5.0. Hypothyroidism emerged as the most common treatment-related AE (52.5%), followed by hypertension (42.5%), diarrhea (37.5%), and fatigue (27.5%). Other notable treatment-related AEs included proteinuria (20.0%), skin pruritus (17.5%), and decreased appetite (17.5%). Among laboratory abnormalities, thrombocytopenia (42.5%) and decreased albumin (40.0%) were most frequently observed, with AST elevation (32.5%), neutropenia (25.0%), ALT elevation (20.0%), and elevated total bilirubin (20.0%) also commonly reported. Grade 3-4 AEs were less frequent, with hypertension and thrombocytopenia (both 12.5%) being the most common severe events. Additional grade 3-4 AEs included hypothyroidism (7.5%), decreased albumin (7.5%), and several events occurring at 5.0% (skin pruritus, diarrhea, and proteinuria). Interestingly, no cases of immunological hepatitis or immune pneumonia were documented throughout the study period.

**Table 5:** Adverse events.

Adverse events, n (%)	Any grade	Grade 1~2	Grade 3~4
Skin and subcutaneous tissue			
Skin rash	1 (2.5%)	1 (2.5%)	
Palmar-plantar erythrodysesthesia	4 (10.0%)	4 (10.0%)	
Skin pruritus	7 (17.5%)	5 (12.5%)	2 (5.0%)
Digestive system			
Gastrointestinal hemorrhage	1 (2.5%)	1 (2.5%)	
Nausea	6 (15.0%)	5 (12.5%)	1 (2.5%)
Diarrhea	15 (37.5%)	13 (32.5%)	2 (5.0%)
Nervous system			
Headache	3 (7.5%)	3 (7.5%)	
Endocrine system			
Hypothyroidism	21 (52.5%)	18 (45.0%)	3 (7.5%)
Systemic symptoms			
Fever	3 (7.5%)	3 (7.5%)	
Fatigue	11 (27.5%)	10 (25.0%)	1 (2.5%)
Peripheral edema	2 (5.0%)	2 (5.0%)	
Kidney and urinary system			
Proteinuria	8 (20.0%)	6 (15.0%)	2 (5.0%)
Cardiovascular system			
Hypertension	17 (42.5%)	12 (30.0%)	5 (12.5%)
Chest and mediastinum			
Dysphonia	4 (10.0%)	4 (10.0%)	
Metabolism and nutrition			
Decreased appetite	7 (17.5%)	7 (17.5%)	
Weight loss	6 (15.0%)	6 (15.0%)	
Blood biochemistry			
ALT elevation	8 (20.0%)	7 (17.5%)	1 (2.5%)
AST elevation	13 (32.5%)	12 (30.0%)	1 (2.5%)
Elevated total bilirubin	8 (20.0%)	8 (20.0%)	
Albumin decreased	16 (40.0%)	13 (32.5%)	3 (7.5%)
Creatinine increased	1 (2.5%)	1 (2.5%)	
Blood routine tests			
Anemia	7 (17.5%)	7 (17.5%)	
Leukopenia	5 (12.5%)	5 (12.5%)	
Thrombocytopenia	17 (42.5%)	12 (30.0%)	5 (12.5%)
Neutropenia	10 (25.0%)	10 (25.0%)	
Immune-related adverse events			
Immunological hepatitis	0		
Immune pneumonia	0		

The data are presented as quantity (percentage).

ALT, alanine transaminase; AST, aspartate transaminase.

## Discussion

Among patients with HCC, arteriovenous shunts occur at rates ranging from 28.8% to 63.2%. These high-flow arteriovenous shunts frequently precipitate severe complications, including esophageal varices, lower extremity edema, ascites, and hepatic encephalopathy. The selection of appropriate therapeutic strategies for HCC with arteriovenous shunts is crucial. Conventional TACE has traditionally served as the standard treatment approach (20). However, outcomes across different medical centers demonstrate considerable variability. The management of these patients presents substantial technical challenges that resist standardization in clinical practice. Furthermore, the risk of non-target embolization remains a significant concern, potentially causing severe deterioration of hepatic function (21).

In recent years, FOLFOX has revitalized HAIC, effectively suppressing tumor growth while improving arteriovenous shunts. With its lower technical requirements, simplified procedures, and consistent outcomes, FOLFOX-HAIC has rapidly expanded across healthcare facilities, benefiting more patients and guiding clinical decisions (22, 23). Emerging data from key phase III studies, including EMERALD-1 (24) and LEAP 012 (25), have convincingly established that patients derive significant clinical advantages from the combined use of interventional therapy with TKIs and ICIs. Feng et al. achieved 35.9% ORR combining TACE with anti-angiogenic therapy and PD-1 inhibitors (26). Our study demonstrated ORR 42.5% as per RECIST v1.1 and 75.0% as per mRECIST, significantly higher than first-line treatments (2%~27.3%) (27), despite all patients having HAVF-a negative prognostic indicator. Our research demonstrated that patients achieved median progression-free and overall survival of 5.5 months (95% CI: 3.9-6.9) and 10.4 months (95% CI: 7.4-13.4), respectively, thus providing further evidence that this combination therapy can significantly ameliorate HAVF, improve tumor regression, and prolong patient survival.

Our study focused specifically on HCC patients with high-flow HAVF, where these shunts primarily result from direct tumor invasion of the portal vein, hepatic vein, or the formation of tumor thrombi (15). In contrast, patients with low-flow HAVF typically present without significant clinical manifestations, and these abnormal shunts often spontaneously resolve following conventional TACE treatment. Therefore, to better evaluate therapeutic efficacy, only HCC patients with high-flow HAVF were included in this investigation. Our findings revealed marked HAVF improvement after 2-3 cycles of triple therapy with complete fistula occlusion documented in most patients, underscoring this combined approach’s effectiveness in HAVF management. Importantly, follow-up evaluations showed no instances of HAVF recanalization, suggesting the durable efficacy of this therapeutic strategy for HAVF. This outcome may be attributed to lesion reduction at the fistula site and subsequent closure, reflecting favorable tumor response to treatment. In contrast, TACE treatment for HAVF shows different outcomes, with higher rates of recanalization and new fistula formation despite initial successful occlusion (17, 28).This divergent outcome likely stems from distinct pathophysiological responses to treatment-TACE procedures create a hypoxic microenvironment that upregulates hypoxia-inducible factors, potentially leading to reopening of shunt connections and tumor recurrence. In contrast, HAIC therapy does not induce such hypoxic cascade reactions, resulting in lower recurrence rates (27).

Our study demonstrates that HAVF significantly impacts the prognosis of patients with hepatocellular carcinoma. Survival analysis revealed marked differences between patients with and without HAVF. The mOS was 4.6 months in patients with HAVF compared to 13.0 months in those without HAVF (P=0.005), while mPFS was 2.1 versus 7.5 months, respectively (P = 0.016). Through comprehensive grading assessment, these findings underscore the critical importance of HAVF in prognostic evaluation of advanced hepatocellular carcinoma, extending beyond previous observations in the literature (29).

Notably, 72.5% of patients achieved HAVF resolution following treatment, representing a clinically meaningful response rate. Multivariate analysis confirmed that HAVF resolution served as an independent protective factor for both overall survival and progression-free survival (HR 0.29, P<0.001; HR 0.20, P=0.027, respectively). This suggests that successful HAVF closure can improve hepatic hemodynamics by reducing portal hypertension, thereby creating more favorable conditions for subsequent therapeutic interventions.

HAVF grades emerged as a crucial prognostic determinant. Patients harboring grade 3 HAVF lesions showed substantially poorer clinical outcomes when compared to those with grade 2 manifestations, establishing HAVF stratification as an independent predictor of overall and progression-free survival. This association likely reflects the more severe hemodynamic disruption associated with higher-grade arteriovenous shunting, leading to progressive hepatic dysfunction.

In our cohort, the overall median survival of advanced HCC patients with HAVF was 10.4 months, a finding of considerable clinical significance. Given that most patients presented with substantial tumor burden and portal vein tumor thrombus, this survival benefit suggests potential advantages of combination therapy in this high-risk patient population.

Furthermore, our study confirmed extrahepatic metastasis as another independent prognostic factor, consistent with established tumor staging principles and clinical experience. Collectively, these findings provide valuable guidance for clinicians in developing individualized treatment strategies and establishing accurate prognostic assessments for this complex and challenging patient population.

In terms of adverse events (AEs), the adverse reactions associated with HAIC combined with TKIs and ICIs in patients with uHCC and high-flow hepatic arteriovenous shunts remained within an acceptable range. The main adverse events comprised mild myelosuppression, abnormal liver function parameters, and hypothyroidism. Notably, no severe complications were detected. These relatively minor adverse reactions can likely be ascribed to the hepatic first - pass effect. This effect reduces the systemic distribution concentrations of chemotherapeutic agents, thereby minimizing the impact on the overall body. All patients who encountered adverse reactions recovered after receiving symptomatic treatment. A comprehensive evaluation indicated that the HAIC treatment protocol utilized in this study demonstrated favorable safety profiles. The adverse reactions were manageable, and the protocol had a high level of clinical acceptability, which makes it a promising approach in the treatment of such patients.

Several methodological limitations warrant acknowledgment in this study. First, as a retrospective analysis, selection bias was unavoidable. Second, the relatively small sample size and limited follow-up period precluded detailed subgroup analyses. Additionally, subsequent immunotherapy or targeted therapy was not evaluated in most patients, which may influence long-term survival outcomes. Future prospective, large-scale randomized controlled trials are needed to validate our findings.

## Conclusion

For patients with uHCC complicated by high-flow hepatic arteriovenous shunts, the combination of HAIC with TKIs and ICIs exhibits promising efficacy and safety characteristics, contributing to the prolonged survival of patients. It represents a treatment option worthy of priority consideration.

## Data Availability

The original contributions presented in the study are included in the article/Supplementary Material. Further inquiries can be directed to the corresponding authors.

## Glossary

HAVF: Hepatic Arteriovenous Fistula
PVTT: Portal vein tumor thrombosis
APS: Arterioportal shunt
TAE: Transcatheter arterial embolization
TACE: Transcatheter arterial chemoembolization
FOLFOX-HAIC: Hepatic arterial infusion of oxaliplatin, fluorouracil, and leucovorin
Don,donafenib: LEN: Lenvatinib
PFS: Progression-free survival
ORR: Objective response rate
mRECIST: Modified Response Evaluation Criteria in Solid Tumors
TKIs: Tyrosine kinase inhibitors
ICIs: Immunotherapy checkpoint inhibitors
DSA: Digital subtraction angiography
ECOG PS: Eastern Cooperative Oncology Group performance status
DEB-TACE: Drug-eluting beads transcatheter arterial chemoembolization
cTACE: Conventional transcatheter arterial chemoembolization
AEs: adverse events
AFP: alpha fetoprotein
CT: Computed tomography
MRI: Magnetic resonance
OS: Overall survival
CR: Complete remission
PR: Partial remission
SD: stable disease
PD: progressive disease
DCR: Disease control rate
IQR: Interquartile range
