# Correction: Triple therapy revolutionizes treatment paradigms for previously untreatable HCC complicated by high-flow hepatic arteriovenous fistulas

**DOI:** 10.3389/fimmu.2025.1681008

**Published:** 2025-08-19

**Authors:** Jinpeng Li, Yuanming Li, Jingtao Zhong, Jiasheng Du, Jiao Chen, Jutian Shi, Lujun Zhao, Jinlong Song

**Affiliations:** ^1^ Department of Radiation Oncology, Tianjin Medical University Cancer Institute and Hospital, National Clinical Research Center for Cancer, Tianjin Key Laboratory of Cancer Prevention and Therapy, Tianjin’s Clinical Research Center for Cancer, Tianjin, China; ^2^ Department of Interventional Therapy I, Shandong Cancer Hospital and Institute, Shandong First Medical University and Shandong Academy of Medical Sciences, Jinan, Shandong, China; ^3^ Interventional Vascular Department, Laizhou Hospital of Traditional Chinese Medicine, Laizhou, Shandong, China; ^4^ Department of Hepatobiliary Surgery, Shandong Cancer Hospital and Institute, Shandong First Medical University and Shandong Academy of Medical Sciences, Jinan, Shandong, China; ^5^ Graduate Department of Shandong First Medical University and Shandong Academy of Medical Sciences, Jinan, China

**Keywords:** hepatocellular carcinoma, high-flow hepatic arteriovenous fistula, hepatic arterial infusion chemotherapy, immune checkpoint inhibitors, tyrosine kinase inhibitors

Author “Lujun Zhao” was erroneously spelled as “Luzhun Zhao”.

The original version of this article has been updated.

